# *Rhodiola rosea* for physical and mental fatigue: a systematic review

**DOI:** 10.1186/1472-6882-12-70

**Published:** 2012-05-29

**Authors:** Sana Ishaque, Larissa Shamseer, Cecilia Bukutu, Sunita Vohra

**Affiliations:** 1 Complementary and Alternative Research and Education (CARE) Program, Department of Pediatrics, Faculty of Medicine and Dentistry, and School of Public Health, University of Alberta, Edmonton, Canada; 2 Clinical Epidemiology Program, Ottawa Hospital Research Institute, Ottawa, Canada; 3 Alberta Centre for Child, Family & Community Research, Edmonton, Canada

## Abstract

**Background:**

*Rhodiola rosea* (*R. rosea*) is grown at high altitudes and northern latitudes. Due to its purported adaptogenic properties, it has been studied for its performance-enhancing capabilities in healthy populations and its therapeutic properties in a number of clinical populations. To systematically review evidence of efficacy and safety of *R. rosea* for physical and mental fatigue.

**Methods:**

Six electronic databases were searched to identify randomized controlled trials (RCTs) and controlled clinical trials (CCTs), evaluating efficacy and safety of *R. rosea* for physical and mental fatigue. Two reviewers independently screened the identified literature, extracted data and assessed risk of bias for included studies.

**Results:**

Of 206 articles identified in the search, 11 met inclusion criteria for this review. Ten were described as RCTs and one as a CCT. Two of six trials examining physical fatigue in healthy populations report *R. rosea* to be effective as did three of five RCTs evaluating *R. rosea* for mental fatigue. All of the included studies exhibit either a high risk of bias or have reporting flaws that hinder assessment of their true validity (unclear risk of bias).

**Conclusion:**

Research regarding *R. rosea* efficacy is contradictory. While some evidence suggests that the herb may be helpful for enhancing physical performance and alleviating mental fatigue, methodological flaws limit accurate assessment of efficacy. A rigorously-designed well reported RCT that minimizes bias is needed to determine true efficacy of *R. rosea* for fatigue*.*

## Background

*Rhodiola rosea (R. rosea)* is a flowering biennial grown in high latitude and altitude regions of the world. It has been a part of traditional medicine systems in parts of Europe, Asia and Russia for centuries. It has been prescribed for cancer and tuberculosis in Mongolia [1], given to newlyweds to boost fertility in Siberia [2], and used by Vikings to increase endurance and physical strength [3]. In Norway, it has even been used as food and hair wash [4].

More recently *R. rosea* has received attention from the scientific community for its potential therapeutic capacity as an adaptogen. Adaptogen are “[most commonly] natural herbal products which are non-toxic in normal doses, produce a non-specific response, and have a normalizing physiologic influence” [5]. Similarly, *R. rosea* has been referred to as an ergogenic aid, i.e. an herb used to enhance physical and mental performance. Common indications pertaining to the adaptogenic and ergogenic capacity of *R. rosea* include performance enhancement, fatigue reduction and alleviation of depression symptoms. Existing reviews suggest a benefit in physical and mental performance attributable to *R. rosea,* however such reviews fail to critically appraise included literature [6,7]*;* some reviews rely on Russian studies that are not accessible in major international databases [8,9]. This systematic review aims to rigourously synthesize and appraise available clinical evidence of the efficacy of *R. rosea* for improving physical and mental performance.

### Active constituents

Active constituents are biologically active components of pharmacological formulations. While it is currently unclear which specific compound(s) in *R. rosea* are active constituents, most preparations of *R. rosea* are standardized to specific levels of marker compounds rosavin, salidroside or both [8]. Rosavin is the only constituent unique the *R. rosea* from the Rhodiola genus; salidroside is common to most other Rhodiola species [10,11]. The naturally occurring ratio of rosavins to salidrosides in *R. rosea* is approximately 3:1 and *R. rosea* preparations have been prepared to reflect this ratio [12].

## Methods

### Search strategy

This systematic review stemmed from a larger review prepared for the Government of Alberta in 2007 for all potential indications of Rhodiola rosea. This review includes a subset of those studies examining *R. rosea* for fatigue. The term “rhodiola rosea” and common synonyms (arctic root, roseroot, rosenroot, golden root and hong jing tian) were used to search the following electronic databases: MEDLINE (1950 – July 2009), Cochrane Evidence Based Medicine databases (1991 – July 2009), EMBASE (1988 – July 2009), Alternative Medicine Database (AMED) (1985 – July 2009), Natural Medicines Comprehensive database (up to July 2009) and The International Pharmaceutical Abstract Database (up to July 2009). The detailed strategy for all databases can be found in the Appendix.

### Inclusion criteria

The inclusion criteria for included studies were as follows: (i) study design: any clinical trial; (ii) population: any clinical human population; (iii) intervention: *Rhodiola rosea* alone or in combination with other compounds; (iv) control: any comparator was considered eligible; (v) outcomes: mental or physical fatigue measured by any means. Where validated instruments were used, this information was collected.

Fatigue can be described as a pervasive sense of tiredness or lack of energy that is not related exclusively to exertion [13]. Fatigue can result due to excess physical or mental activity, sleep deprivation, and poor diet or range of medical conditions including infection, and cardiovascular, metabolic, connective tissue and endocrine disorders [13]. Division of mental and physical fatigue is arbitrary and often the two cannot be differentiated [13-15]. As such, studies describing physical or mental fatigue were both eligible for inclusion.

Due to the natural geographical distribution of the *R. rosea* plant (i.e. Scandinavian countries, Russia and parts of northern Asia), both English and non-English literature were considered. Non-English studies were translated into English.

### Study selection and data extraction methods

Two authors (LS and SI) independently screened studies identified using the search strategy for inclusion, first on the basis of title and abstract and of those that were relevant, the full texts were screened for eligibility. The same two authors independently extracted data from each study using pre-developed data extraction forms. Discrepancies between reviewers were discussed and resolved by consensus. Authors of included studies were contacted to confirm and answer questions about the data.

### Data extraction items

Items for which data were extracted include: publication year, country of publication, study design, population description, diagnostic criteria, age, intervention description, % salidrosides, % rosavins, control description, dose, duration, frequency, run-in period length, washout period length, follow-up period, number of participants randomized and analysed in each group, effect measures and measures of precision, outcome measurement tools, author’s conclusions, description and number of adverse events in each group.

### Risk of bias assessment

Two reviewers (LS and SI) independently assessed the risk of bias of each trial, following the domain-based evaluation endorsed by the Cochrane Collaboration [16]. The domains are as follows: randomization, concealment of allocation, blinding (of participants, personnel and outcome assessors), incomplete outcome data (whether investigators used an intention-to-treat analysis), selective outcome reporting, and other factors.

In the first three domains, and answer of “Yes” means a low risk of bias, “No” means there is a high risk of bias and “unclear” means there is an uncertain risk of bias (likely due to poor reporting). In the latter three domains, responses mean the opposite - “yes” means high risk of bias and “no” mean low risk of bias.

### Analysis plan

We planned to report continuous outcomes as changes from baseline and where possible, to compare the difference between *R. rosea* and control as mean differences (MD) with 95% confidence intervals (CI). We planned to convert dichotomous outcomes into risk ratios and 95% CIs. Meta-analysis was planned where more than one study provided data for a single outcome.

## Results

### Description of included studies

Two hundred and six unique articles were identified from the search and 11 met final inclusion criteria. Of those, 10 were described as randomized controlled trials (RCT) [17-26] and one was a controlled clinical trial (CCT) [24]. Six studies examined the effect of *R. rosea* on physical performance and five assessed mental fatigue. None of the studies examining *R. rosea* examining physical or mental fatigue measured outcomes consistently - no two studies reported the same outcomes. As such, meta-analysis could not be performed. Included studies are described in text below and Table 1.

**Table 1:** Rhodiola rosea summary of clinical evidence

**PHYSICAL PERFORMANCE**				
***R. rosea *** **single ingredient versus placebo**				
**Study ID**	**Design**	**Population**	**Intervention/Control**	**Outcome(s)**
Abidov 2004	DB RCT	36 male and female non-smokers between 21–24 y.o.a.	**Int:** 340 mg RR extract containing 30 mg active RR (including rosavin)	CRP blood-levels 5 h and 5 days after exercise were less than placebo and control.
(Russia)				
			**Con(1):** placebo	
			**Con(2):** no intervention;	CK levels in blood same across groups after 5 hours, but reduced after 5 days in only *R. rosea * group.
			***Regimen:*** for 30 days before and 6 days after exhausting physical exercise.	
Walker 2007	DB CO RCT	12 resistance-trained males (19–39 y.o.a.)	**Int:** 1500 mg/day RR for 3 days before exercise test + 1000 mg on day of test **Con:** placebo;	No significant differences between groups in measures of ATP kinetics and exhaustion.
(US)				
			***Regimen:*** RR followed by 7–14 days washout and same dosing regimen of placebo OR treatment in reverse order	
***R. rosea *** **Plus starch versus placebo**				
De Bock 2004a	DB CO RCT	24 healthy physically active male (21.8 ±0.3 y.o.a) and female (20.2 ± 0.3 y.o.a.) students	**Int:**100 mg RR +250 mg starch	RR ‘significantly’ delayed time to exhaustion, peak O_2_ uptake and CO_2_ output.
(Belgium)				
			**Con:** 350 mg starch	
			***Regimen:*** 2 days of RR, 5 days washout, 2 days placebo	
De Bock 2004b	DB CCT		**Int:**100 mg RR +250 mg starch	significantly higher blood lactate levels after 4-wk intake.
(Belgium)				
			**Con:** 350 mg starch	
			***Regimen:*** RR or placebo twice/day over 4 weeks	Overall, no change in muscle strength, speed of limb movement, reaction time, sustained attention.
***R. rosea *** **plus Cordyceps versus Placebo**				
Earnest 2004 (US)	DB RCT	17 male competitive cyclists	**Int:** loading dose of 6 capsules/day for 4 days (every three capsules contain 1000 mg *Cordyceps sinensis* + 300 mg RR) then maintenance dose of 3 capusles/day for 11 days	No significant difference between or within Tx groups in peak and subpeak exercise variables.
		int: 31.6 ± 2.8 (SE) y.o.a.		
		con: 30.5 ± 2.2 y.o.a.		
			**Con:** identical placebo	
Colson 2005 (US)	DB RCT	8 males between 18–34 y.o.a.	**Int:** loading dose of 6 capsules/day for 6 days (every three capsules contain 1000 mg *Cordyceps sinensis* + 300 mg RR) then maintenance dose of 3 capsules/day for 7 days	After pre-post endurance test no significant difference between intervention and placebo in muscle tissue oxygen saturation
			**Con:** identical placebo	
**MENTAL PERFORMANCE**				
**Study ID**	**Design**	**Population**	**Intervention/Control**	**Outcome(s)**
Olsson 2008	DB RCT	60 male and female (20–55 y.o.a) with fatigue syndrome	**Int:** 4 Verum tablets/day for 28 days (each tablet contain 144 mg Rhodiola extract SHR-5)	Significant improvement in fatigue scores (measured by Pines burnout scale), and significant improvement in two of five CCPT II indices
			**Con:** identical placebo	
		int: 41.0 ± 7.9 y.o.a		
		con: 42.1 ± 8.5 y.o.a.		
Darbinyan 2000	DB CO RCT	56 male and female physicians on night duty	Group A: Standardized extract of 170 mg RR for 2 weeks; 2 week Washout; 2 weeks identical placebo. Group B: treatment in reverse order	Significant improvement in total fatigue score after two weeks on RR;
(Armenia)				
		Group A: 25.5 ± 3.8 y.o.a.		
		Group B: 27.3 ± 2.9 y.o.a.		
Shevstov 2003	DB RCT	121 male military cadets 19–21 y.o.a.	Int: 41 subjects – 2 capsules (185 mg each) RR.	Total antifatigue index scores significantly lower in both RR groups than placebo (p < 0.0001).
(Russia)				
			20 subjects – 3 capsules RR	
				“statistically significant beneficial physiological effect in the RR groups versus the placebo group”
			Con: 40 subjects – identical placebo	
			20 subjects – untreated control	
Spasov 2000	DB RCT (pilot study)	40 Male students from India 17–19 y.o.a.	Int: 2 x 50 mg RR tablets twice/day for 20 days	Non-significant improvement in physical work and mental capacity. Significant improvement in general well-being (p < 0.05), mental fatigue (p < 0.01).
(Russia)				
			Con: identical placebo	
Spasov 2000 translated (Russia)	RCT	60 male students from India (17–18 y.o.a.)	Int: 660 mg/day for 20 days RR; Con(1): placebo; Con(2): nothing	Measures of subjective self-evaluation, psychological fatigue, situational anxiety, motivation, precision of motor function, process of excitement and need for rest are significantly different within RR group (<0.05). Mean changes in mental work capacity and neuromotor function are not significantly different between groups.

Abbreviations: *RR Rhodiola rosea*, *DB* double-blind, *RCT* Randomized controlled trial, *CCT* Controlled clinical trial, *CO* cross-over, *Int* Intervention, *Con* Control, *DO* Drop-outs, *AE* Adverse events.

### Risk of bias in trials of rhodiola rosea

**Figure 1:**
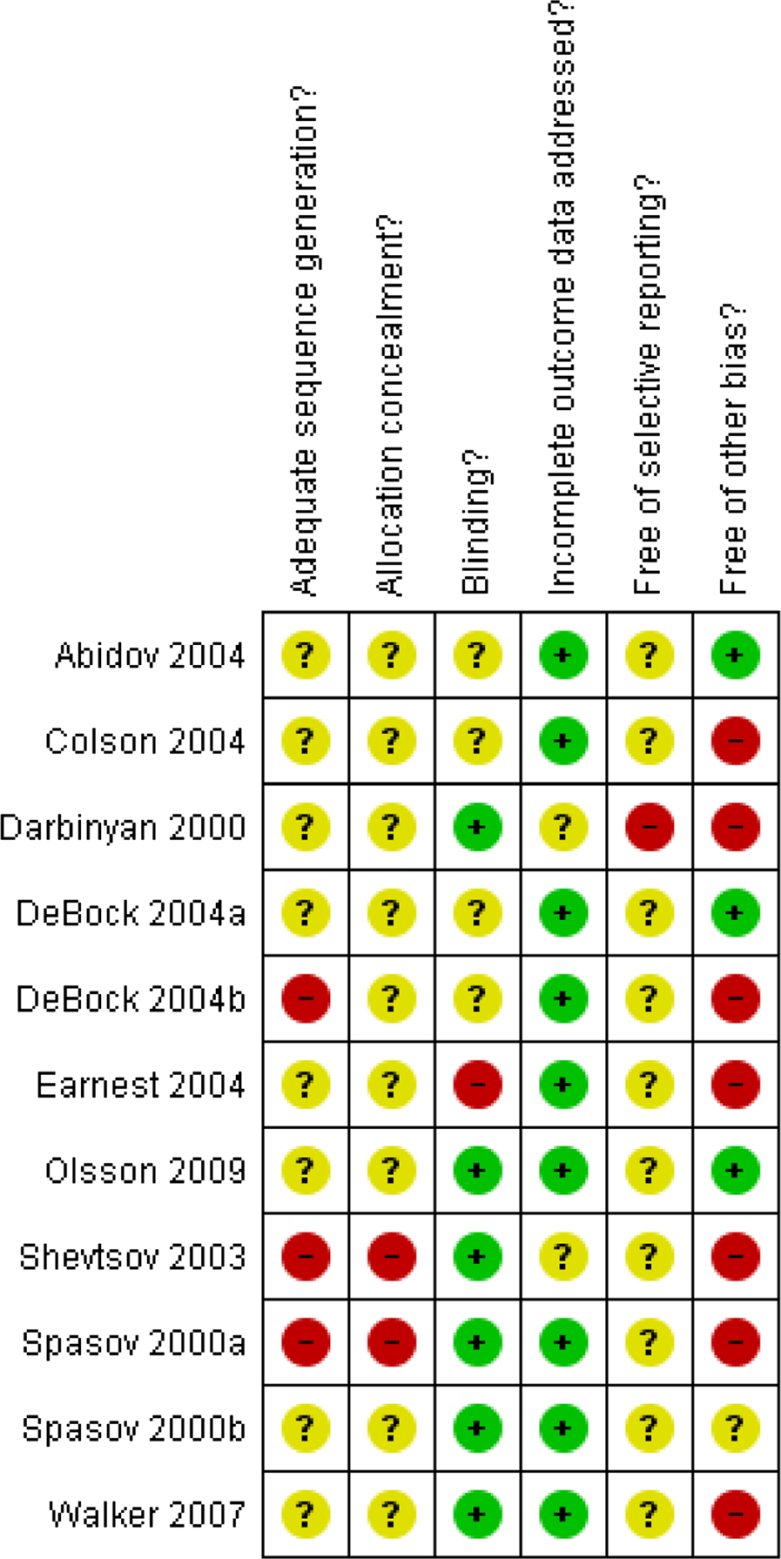
Risk of bias assessment in included studies.

As can be seen from Figure 1, the majority of studies in each domain have an ‘unclear’ risk of bias in almost every domain due to how they were reported. None of the included studies are free of plausible bias (Figure 1), which raises potential concern about the validity of their results. In the domain of “other risk of bias”, six studies reporting non-significant results have a low risk of bias due to lack of sample size calculation [18,19,21,25,26], one calculated a sample size but did not specify a primary outcome [24] and three reported the use of outcome measurement tools that are not validated [19,24].

### Physical fatigue

Five RCTs and one CCT of *R. rosea* for enhancing physical performance were identified. Two trials examine a *R. rosea*-only supplement, two examined *R. rosea-strach* combined, and another two evaluate *R. rosea* combined with cordyceps.

### *R. Rosea* as single ingredient versus placebo

A three arm double blind RCT compared the effect of *R. rosea* (as a single ingredient) to placebo, or nothing [17]. The study examined muscle recovery in 30 adults by measuring C-reactive protein (CRP) and creatinine kinase (CK) levels in blood. Subjects underwent an exhausting physical exercise test on day 30 which consisted of cycling at 20 W on a bicycle ergometer with power increased by 10 W/min until volitional exhaustion (i.e. subject could no longer pedal at 60 rpm). Findings indicate that *R. rosea* significantly lowered CRP levels at 5 hours and 5 days after the test (*p* < 0.05) but that CK levels were not significantly different between groups. Adverse events were not reported.

A double-blind cross-over RCT examined the effect of *R. rosea* on exercise performance in twelve male subjects [25]. Subjects received *R. rosea* or identical placebo for 3 days before outcomes were measured by an exercise test and another dose on the day of the test. A wash-out period of 7 to 14 days separated cross-over to the opposite treatment. The primary outcome was muscle recovery measured by ATP levels and secondary outcomes were time to exhaustion and perceived exertion; all outcomes were measured at baseline, during the exercise test and during recovery. There was no significant difference between groups in P_i_, phosphocreatine and ATP levels, time to exhaustion and perceived exhaustion. Adverse events and drop-outs were not mentioned.

### *R. Rosea* plus starch versus starch alone

One cross-over RCT and one CCT described in a single report examined the acute and long-term effects, respectively, of *R. rosea* on exercise performance [20]. In both studies, endurance capacity was the primary outcome and muscle strength, speed of limb movement, reaction time and sustained attention were secondary outcomes.

In the first study on acute effects, *R. rosea* combined with starch or placebo was taken on each of 2 days [20]. One hour after ingestion on each day, outcomes were measured while subjects underwent a physical functioning test. After a five day washout period, subjects switched to the alternate treatment and performed the same tests. Baseline measurements were not taken. Three out of six parameters of endurance capacity (time to exhaustion, O_2_ uptake and CO_2_ output) significantly improved (*p* < 0.05) in the *R. rosea* group. There was no difference between groups in any secondary outcomes. After five days, authors stated that 12 subjects were reassigned to intervention and control groups for the long-term evaluation study. The long term study evaluated subjects receiving same intervention and control as in the acute study twice per day over a four week period [20]. The same outcomes as in the acute study were measured. Long term supplementation produced no significant difference in any outcomes between treatment groups; one participant on *R. rosea* dropped out during long term supplementation for medical reasons unrelated to the study protocol (reason not stated). One subject with strong headaches during acute supplementation and one with minor headaches during long term supplementation were both on placebo. One subject experienced a minor headache and another had insomnia during long term supplementation of *R. rosea*. It is unclear why the long-term study was not randomized.

### *R. Rosea* plus cordyceps versus placebo

Two double blind RCTs conducted evaluate the effect of *R. rosea* combined with other herbs on exercise performance [18,21]. Both studies were conducted by the same group of authors using slightly different protocols and populations. In both studies, intervention capsules were described as every 3 capsules containing 300 mg of *R. rosea (s*tandardized to 3.0% rosavins and 2.5% salidrosidesminimum), 1000 mg of *Cordyceps sinensis*, a Chinese herb reported to improve circulation [27], and 800 mg of the manufacturers ‘proprietary blend’ of substances (undisclosed).

In one of the RCTs, 17 male were randomly assigned to either the *R. rosea*-containing formulation or placebo for 15 days [21]. Subjects took six capsules per day for 4 days (loading dose) then three capsules per day 11 days (maintenance dose). Endurance capacity was measured by multiple parameters including peak CO_2_ output, power output, time to exhaustion and peak heart rate, which were measured at the beginning and end of the study period. The authors found that the herbal formulation did not have any significant effect on exercise endurance or capacity. Adverse events were not reported.

The second study involving eight male cyclists randomized to either *R. rosea*-containing formulation (33.0 ± 12.6) years) or placebo (23.8 ± 2.9 years), followed the same protocol as above, however the study period was only 13 days – 6 days of the loading dose and 7 days of the maintenance dose [18]. Respiratory parameters were measured in the participants. This study also found no significant difference in outcomes between groups. There were no drop outs; adverse events were not mentioned.

### Mental fatigue

A double blinded RCT assessed the efficacy of a *R. rosea* extract, SHR-5, for stress related fatigue [22]. Sixty subjects were randomized to receive 576 mg of *R.* rosea preparation or placebo per day for 28 days. Mental fatigue, measured by the Pines burnout scale, was the primary outcome. Other outcomes evaluated were depression (Montgomery-Asberg Depression Rating Scale, MADRS), quality of life (Medical Outcomes Study Short form 36-item questionnaire, SF-36), attention (Conners’ Computerized Continuous Performance Test II, CCCPT II) and “anti-fatigue” effect (saliva cortisol response after awakening). All outcomes were measured before and after the treatment period. The Pines burnout scale scores (*p* = 0.047) and two out of five indices of CCCPT II (*p* = 0.02, *p* = 0.001) improved in favour or *R. rosea*. While investigators conclude that the treatment appears to have beneficial effect, they report excluding follow-up data for at least 5 participants due to physical loss of data and protocol deviations. Per-protocol analyses (i.e. analysis of only participants who followed the protocol for the entirety of the study) may overestimate treatment effect if the reasons for incomplete data are related to the treatment effect [28,29] – in this case, it is not explicitly stated what “protocol deviations” occurred. No adverse effects occurred during the study period.

*R. rosea* for non-specific fatigue was evaluated in a double-blind crossover RCT in 56 Armenian physicians [19]. Participants were randomized to either 170 mg *R. rosea* (standardized to 2.6% salidroside) or placebo. The study period lasted for two weeks followed by a two-week wash-out period, after which participants were crossed over for two weeks. The primary outcome was fatigue, measured using a fatigue index developed for use in this study; the tool does not appear to be validated. Measurements were carried out before and after the treatment period. Authors state that they found a significant improvement in the fatigue index after two weeks of *R. rosea* supplementation, but only present data for the 5 individual test scores. Since we are unable to replicate and confirm their analysis, findings of this study must be interpreted as inconclusive. Authors indicate that no adverse events occurred; whether or not anyone dropped out of the study was not reported.

A double-blinded RCT conducted in Russia evaluated the effect of two different single doses of *R. rosea* on mental fatigue [23]. Subjects were randomized to take *R. rosea* or placebo. A non-treatment group was also included, however subjects were not randomized into this group and comparisons against this group will not be considered in this review. The intervention was taken at 4:00 am while participants were on an overnight shift. Capacity for mental work, measured using a fatigue index of unknown origins and pulse pressure and rate were evaluated before night duty and one hour after taking the study medication. A self-report questionnaire evaluating general well-being was completed after taking the study medication. The fatigue index was comprised of three parameters: visual perception, short-term memory and perception of order. Improvements in favour of both doses of *R. rosea* were apparent in the fatigue index (*p* < 0.001); no significant differences between groups occurred for other outcomes. The method of randomization was unclear. One subject in the placebo group experienced hypersalivation; whether or drop-outs occurred was not reported.

A double-blinded RCT pilot study examined the effect of a repeated low dose of *R. rosea* on foreign students’ mental and physical well-being during their examination period [24]. Subjects were randomized into 2 groups to receive either 100 mg *R. rosea* once per day or identical placebo for 20 days. Hand-eye coordination (maze test), motoric speed (tapping test), mental work capacity (correction of text test), fatigue and well-being (self-evaluation questionnaire), heart rate and physical work capacity (bicycle ergometer test) were assessed. Significant improvements were observed in hand-eye coordination (*p* < 0.01), mental fatigue and general-well being (*p* < 0.01) in favour of *R. rosea*. Students on placebo had a significantly higher heart rate (*p* < 0.05). Drop-outs and adverse events were not reported by authors.

Another RCT conducted by the same group examined 60 male students in their first year of study at a Russian high school [26]. Students were randomized into 3 groups to receive either of Rhodaxon (*R. rosea* extract with no ethyl alcohol per day; proportions of active constituents not given), placebo or nothing for 20 days. Participants underwent the same tests for mental and physical capacity as above as well as a psychophysiological test [Lusher test [30]] to determine level of anxiety, psychological fatigue and need to rest. A comparative analysis between groups was not conducted leaving the effect of *R. rosea* indeterminable. Adverse events and drop-outs were not reported.

### Adverse effects

Out of 446 subjects examined in the 11 included clinical studies, five adverse events were mentioned in three studies. Two subjects on 200 mg of *R. rosea* over a 4-week period each experienced a minor and serious headache [24]; one subject on placebo over a 2-day treatment period experienced a minor headache and another had insomnia [20]. Another subject on placebo experienced hypersalivation [23]. There appear to be few side effects associated with *R. rosea* supplementation; those identified are of a mild nature.

## Discussion

Even though 10 of the 11 included studies are RCTs, there is insufficient evidence to evaluate the effectiveness of Rhodiola rosea on physical or mental fatigue. While RCTs are the modern day gold standard for assessment of efficacy of medical interventions, none of the included RCTs appeared to be compliant with the CONSORT (*Con*solidated *S*tandards of *R*eporting *T*rials) Statement, an internationally agreed-upon set of RCT reporting guidelines [31]. In addition, appraisal using the Cochrane Collaboration’s new tool for assessing bias in RCTs, indicates a largely unclear or high risk of bias within included studies.

Of the five RCTs of *R. rosea* for enhancing mental performance identified, three indicated that the herb may be effective in improving overall health in a mentally fatigued population. However, two of these studies did not appear to use validated measures of fatigue, making the validity of their findings difficult to assess. The remaining studies offered inconclusive or negative results. Further rigorous RCTs are required in order to determine the effect of *R. rosea* on mental fatigue.

*R. rosea* has demonstrated a very low occurrence of side effects demonstrating a low clinical toxicity. Although no contraindications with other herbal or prescription medications have been identified, it is important to consider that *R. rosea* may have an additive effect with other substances exhibiting stimulant properties [32]. Like many natural health products, the likelihood of adequate reporting of adverse events may be lower than conventional medications [33].

Clinical studies report *R. rosea*-only products ranging in dose from 50 mg to 660 mg per capsule, to a maximum of 1500 mg/day, suggesting a large margin of safety. Studies reporting a positive effect of *R. rosea* on physical performance reported doses of 200 mg/day and 680 mg/day and those reporting a positive effect on mental fatigue reported doses between 100–576 mg/day.

### Limitations

It is not possible to know the sole effect of *R. rosea* from combination interventions. However our goal was to be comprehensive in our approach, and these studies were included in case they provided interesting hypothesis generating questions for future research.

No studies in this review specifically included pediatric populations or pregnant or lactating women. As well, pregnant or lactating women were explicitly excluded from the majority of clinical studies. No indications have been identified which are specifically relevant to either of these populations, nor have reports of toxicity or adverse events. Overall, due to the paucity of clinical studies including these populations, no dosage recommendations can be made until further studies have been conducted which evaluate the safety and/or toxicity of *R. rosea* in children and pregnant or lactating women.

## Conclusion

The current evidence for efficacy of *R. rosea* is contradictory and inconclusive. Methodologically rigorous RCTs must be designed to overcome these serious threats to internal validity. Such studies will help inform policy-makers, health care providers, and the public about the efficacy *R. rosea* supplementation for physical and mental performance.

## Appendix

### Medline search

1. rhodiola rosea.mp. [mp = title, original title, abstract, name of substance word, subject heading word]

2. roseroot.mp. [mp = title, original title, abstract, name of substance word, subject heading word]

3. golden root.mp. [mp = title, original title, abstract, name of substance word, subject heading word]

4. arctic root.mp. [mp = title, original title, abstract, name of substance word, subject heading word]

5. king's crown.mp. [mp = title, original title, abstract, name of substance word, subject heading word]

6. rosenroot.mp. [mp = title, original title, abstract, name of substance word, subject heading word]

7. rodia riza.mp. [mp = title, original title, abstract, name of substance word, subject heading word]

8. lignum rhodium.mp. [mp = title, original title, abstract, name of substance word, subject heading word]

9. sedum rhodiola.mp. [mp = title, original title, abstract, name of substance word, subject heading word]

10. sedum rosea.mp. [mp = title, original title, abstract, name of substance word, subject heading word]

11. hong jing tian.mp. [mp = title, original title, abstract, name of substance word, subject heading word]

12. exp Rhodiola/

13. or/1-12

14. limit 13 to humans

### Cochrane database of systematic reviews (the whole suite)

1. Rhodiola/

2. (roseroot or golden root or arctic root).mp. [mp = title, original title, abstract, mesh headings, heading words, keyword]

3. (king's crown or rosenroot or rodia riza or lignum rodium or lignum rhodium or sedum rhodiola or sedum rosea or hong jing tian).mp. [mp = title, original title, abstract, mesh headings, heading words, keyword]

4. 1 or 2 or 3

5. humans/

6. 4 and 5

### Amed

1. exp Rhodiola/

2. rhodiola rosea.mp.

3. roseroot.mp.

4. golden root.mp.

5. arctic root.mp. [mp = abstract, heading words, title]

6. king's crown.mp. [mp = abstract, heading words, title]

7. rhodiola.mp. [mp = abstract, heading words, title]

8. rosenroot.mp. [mp = abstract, heading words, title]

9. rodia riza.mp. [mp = abstract, heading words, title]

10. lignum rhodium.mp. [mp = abstract, heading words, title]

11. sedum rhodiola.mp. [mp = abstract, heading words, title]

12. sedum rosea.mp. [mp = abstract, heading words, title]

13. hong jing tian.mp. [mp = abstract, heading words, title]

14. 4 or 1 or 3 or 7 or 12 or 2**IPA**

1. rhodiola.mp. [mp = title, subject heading word, registry word, abstract, trade name/generic name]

2. roseroot.mp. [mp = title, subject heading word, registry word, abstract, trade name/generic name]

3. golden root.mp. [mp = title, subject heading word, registry word, abstract, trade name/generic name]

4. arctic root.mp. [mp = title, subject heading word, registry word, abstract, trade name/generic name]

5. king's crown.mp. [mp = title, subject heading word, registry word, abstract, trade name/generic name]

6. rosenroot.mp. [mp = title, subject heading word, registry word, abstract, trade name/generic name]

7. rosen root.mp. [mp = title, subject heading word, registry word, abstract, trade name/generic name]

8. rodia riza.mp. [mp = title, subject heading word, registry word, abstract, trade name/generic name]

9. lignum rhodium.mp. [mp = title, subject heading word, registry word, abstract, trade name/generic name]

10. sedum rhodiola.mp. [mp = title, subject heading word, registry word, abstract, trade name/generic name]

11. sedum rosea.mp. [mp = title, subject heading word, registry word, abstract, trade name/generic name]

12. hong jing tian.mp. [mp = title, subject heading word, registry word, abstract, trade name/generic name]

13. or/1-12

14. limit 13 to human

### Embase

1. exp Rhodiola Extract/or exp Rhodiola/or exp Rhodiola Rosea Extract/

2. exp rhodiola rosea/or rhodiola rosea.mp.

3. roseroot.mp.

4. golden root.mp.

5. arctic root.mp.

6. king's crown.mp.

7. rosenroot.mp. [mp = title, abstract, subject headings, heading word, drug trade name, original title, device manufacturer, drug manufacturer name]

8. rosen root.mp. [mp = title, abstract, subject headings, heading word, drug trade name, original title, device manufacturer, drug manufacturer name]

9. rodia riza.mp. [mp = title, abstract, subject headings, heading word, drug trade name, original title, device manufacturer, drug manufacturer name]

10. lignum rhodium.mp. [mp = title, abstract, subject headings, heading word, drug trade name, original title, device manufacturer, drug manufacturer name]

11. sedum rhodiola.mp. [mp = title, abstract, subject headings, heading word, drug trade name, original title, device manufacturer, drug manufacturer name]

12. sedum rosea.mp. [mp = title, abstract, subject headings, heading word, drug trade name, original title, device manufacturer, drug manufacturer name]

13. hong jing tian.mp. [mp = title, abstract, subject headings, heading word, drug trade name, original title, device manufacturer, drug manufacturer name]

14. or/1-13

15. limit 14 to human

## Competing interest

SV receives salary support from Alberta Innovates-Health Solutions. None of the authors have a personal or financial competing interest.

## Authors’ contributions

All authors read and approved the final manuscript. Author contributions were as follows: SV conceived of the review, LS, CB and SV designed the review, LS, CB and SI conducted the review (performed screening, data extraction, risk of bias assessment); LS analysed dated; SI, LS and CB contributed to the writing of the review; SV provided guidance and supervision and has primary responsibility for final content. All authors critically revised the manuscript for important intellectual content.

## Financial disclosure

This project was funded by Canadian Foundation for Innovation (New Initiatives fund 2004/2005), Alberta Value-Added Corporation (AVAC), Alberta Crop Industry Development Fund (ACIDF), Alberta Agriculture and Rural Development (AARD). Funders did not play any role in the concept and design of the study.

## Pre-publication history

The pre-publication history for this paper can be accessed here:

http://www.biomedcentral.com/1472-6882/12/70/prepub
